# COVID-19-related anxiety and the role of social media among Canadian youth

**DOI:** 10.3389/fpsyt.2023.1029082

**Published:** 2023-06-05

**Authors:** Soyeon Kim, Kimberly D. Belfry, Jennifer Crawford, Arlene MacDougall, Nathan J. Kolla

**Affiliations:** ^1^Waypoint Centre for Mental Health Care, Waypoint Research Institute, Penetanguishene, ON, Canada; ^2^Psychiatry and Behavioural Neurosciences, McMaster University, Hamilton, ON, Canada; ^3^Faculty of Health Sciences, Ontario Tech University, Oshawa, ON, Canada; ^4^Psychiatry and Epidemiology & Biostatistics, Western University, London, ON, Canada; ^5^Centre for Addiction and Mental Health, University of Toronto, Toronto, ON, Canada

**Keywords:** social media, screen time, COVID-19, youth, COVID-19-related anxiety

## Abstract

**Background:**

Current literature indicates that safety measures, including lockdowns during the COVID-19 pandemic, severely disrupted our lifestyle, marked by increased screen time. The increased screen time is mostly associated with exacerbated physical and mental wellbeing. However, the studies that examine the relationship between specific types of screen time and COVID-19-related anxiety among youth are limited.

**Methods:**

We examined the usage of passive watching, social media, video games, and educational screen time and COVID-19-related anxiety at the 5-time points (Early-Spring 2021, Late-Spring 2021, Fall 2021, Winter 2022, and Spring 2022) among youth in Southern Ontario, Canada (*N* = 117, mean age = 16.82, male = 22%, non-White = 21%) and investigated the role of 4 types of screen time in COVID-19 related anxiety. COVID-related anxiety was measured using the Coronavirus Anxiety Scale (CAS). Descriptive statistics examined the binary relationships between demographic factors, screen time, and COVID-related anxiety. Also, partially adjusted and fully adjusted binary logistic regression analyses were conducted to examine the association between the types of screen time and COVID-19-related anxiety.

**Results:**

During the late Spring of 2021, when the provincial safety restrictions were most stringent, screen time was the highest among the 5 data collection time points. Further, adolescents experienced the highest COVID-19-related anxiety during this period. On the other hand, young adults experienced the highest COVID-19-related anxiety during Spring 2022. In a partially adjusted model (accounting for other types of screen time), engaging in 1–5 h per day on social media increased the likelihood of experiencing COVID-19-related anxiety compared to those who spend less than 1 h per day (OR = 3.50, 95%CI = 1.14–10.72, *p* < 0.05). Other types of screen time was not significantly associated with COVID-19-related anxiety. In a fully adjusted model (accounting for age, sex and ethnicity besides four types on screen time), 1–5 h per day of social media remained significantly associated with COVID-19-related anxiety (OR = 4.08, 95%CI = 1.22–13.62, *p* < 0.05).

**Conclusion:**

Our findings suggest that COVID-19-related anxiety is associated with youth engagement in social media during the COVID-19 pandemic. Clinicians, parents, and educators should work collaboratively to provide developmentally appropriate approaches to reduce the negative social media impact on COVID-19-related anxiety and promote/foster resiliency in our community during the recovery period.

## 1. Introduction

The first case of COVID-19 was reported on January 23, 2020, in Ontario, Canada (1). The Ontario provincial government responded to various waves of the virus over the following 2 years by implementing public health measures. This included public health measures limiting individuals’ contact, activities, and movement and the government-mandated closure of non-essential businesses, all indoor recreational programs, public libraries, theatres, and all outdoor recreational spaces, including parks and walking trails; and the transition from in-person learning to virtual education in grade schools (1–3). While some of these public health safety restrictions were lifted by June 22, 2020, ongoing changes to these restrictions have continued as there are still intense pressures on the healthcare system (e.g., backlogs from surgeries) (4, 5). As a result, people are still struggling with various mental health challenges, including COVID-19-related anxiety (i.e., dysfunctional anxiety associated with the COVID-19 crisis) (6, 7).

Current literature indicates that public health measures during the COVID-19 pandemic severely disrupted youth’s lifestyle, marked by increased time spent in front of screens, including smartphones, TV, and computers (i.e., screen time), in particular, use of social media (8–12). For example, daily screen time doubled to over 5 hours during the pandemic compared to pre-pandemic usage levels among young adults in the US (10). Similarly, 30.8% of Canadian youth reported using social media for 5 or more hours daily during the pandemic (13). With the public safety measures in place, youth may have shifted their way of connecting using internet-based technologies, such as social media, as an alternative platform to play and socialize with friends and families (14). Staying socially and emotionally connected with peers and family through internet-based technology is important during the pandemic for social support and may alleviate feelings of loneliness and isolation (14, 15).

While screen time and social media platforms are one of the limited options for youth to connect during the pandemic, the increased social media screen time is associated with deterioration in many mental health domains among youth during the pandemic (8, 12, 16–20). For instance, a multi-national cross-sectional study (United States, United Kingdom, and Australia) on adolescents (*N* = 3,810) reported that frequent use of social media was associated with loneliness and a high level of emotional distress during the pandemic (21). Canadian adolescents also expressed concerns about the COVID-19 pandemic-related peer relationship and schooling issues (11). Notably, US (22) and Canadian adolescents (11) who spent more time on social media were associated with more COVID-19-related distress, loneliness and depression. Similarly, those exposed frequently to social media were positively associated with high odds of anxiety and a combination of depression and anxiety (16).

Given the possibility of continued screen time trends beyond the pandemic and extensive findings on the adverse impact on mental health, more research is warranted to clarify the role of screen time and social media on youth mental health. For example, studies investigating the role of specific types of screen time are limited. There is a potential overlap of screen time usage (e.g., one can multi-task texting and watching Netflix®), and the impact of each type of screen time on mental health may be different. Hence, it is crucial to delineate the role of different screen time types in mental health in the context of the pandemic. A recent study reported a stronger impact of active screen time (e.g., online games, social media) on psychosomatic complaints than passive screen time (e.g., watching TV) during the pandemic (20). However, further differentiation of screen time types commonly used during the pandemic, such as social media, online games, passively watching (e.g., Netflix®, T.V.), and education (e.g., online learning), is warranted. Furthermore, the role of screen time on COVID-19-specific anxiety is less investigated. As the pandemic is taking its course and safety measures are being adjusted based on its impact, it is important to understand how youth feel about the pandemic (e.g., COVID-19-related anxiety) and the factors associated with it to support them effectively.

In the current study, we examined the usage of passive watching, social media, video games, and educational screen time and COVID-19-related anxiety at five-time points (Early-Spring 2021, Late-Spring 2021, Fall 2021, Winter 2022, and Spring 2022) during the pandemic among youth in Southern Ontario, Canada. Further, we investigated the impact of the social media on COVID-19-related anxiety among Canadian youth during the pandemic. We hypothesized that the frequent use of social media would be associated with increased COVID-19-related anxiety among youth during the pandemic.

## 2. Methods

### 2.1. Procedure

This cross-sectional study is part of the study “Mindfulness and Social–Emotional Learning in Youth,” examining the impact of a virtually-delivered mindfulness intervention on social–emotional competence in youths who engage in screen time. From April 2021 to April 2022, community youths were recruited from central- and north-central Ontario using digital flyers and word-of-mouth. The final sample (*N* = 117) used in this study consisted of the pre-survey of five cohorts (Early-Spring 2021, Late-Spring 2021, Fall 2021, Winter 2022, and Spring 2022), each recruited for up to 8-weeks. A complete pre-survey and informed consent were required for program participation (online mindfulness intervention). Informed consent and survey responses were collected using REDCap®, a secure online data repository system. The Institutional Research Ethics Board approved all components of this study (HPRA# 21.03.02).

### 2.2. Participants

All youth aged 18 to 25 will be invited to participate, with the exception of those who do not speak or understand English. The average participant age was 16.8 years old (SD 3.7; range 12–25), 67% of the participants were categorized as “adolescent” (12–17 years). In terms of ethnicity, 77% of participants were White, 8% were Asian, and 6% were First Nations or Metis. The sample was 78% female with 65% identifying as girl/woman (boy/man = 22%; other = 13%). Participants were recruited in central- and north-central Ontario (73% North Simcoe Muskoka region, 22% Guelph-Wellington, and 5% Haliburton region).

### 2.3. Measures

#### 2.3.1. Demographics

Demographic information, including age, sex assigned at birth, and ethnicity were collected. Participant age, sex assigned at birth, and ethnicity were dichotomized as “adolescent” (12–17 years) or “young adult” (≥18 years), “male” or “female,” and “White” or “Non-White (Asian, First Nations or Metis)” respectively.

#### 2.3.2. Screen time

Participants were asked to report how many hours per day, on average, they dedicate to screen time across four types of usage (passive watching, social media, video games, and education). Passive screen time was defined as “[watching] TV, movies or videos, including YouTube® for pleasure”; social media as “[time spent] on social media (i.e., Facebook®, Instagram®, and Snapchat®, etc.)”; video games as, “[playing] video games (online and/or offline)”; and education as, “[use of] an electronic device (i.e., computer, laptop, tablet) for educational purposes (i.e., schooling).” Response choices for all four types of screen time were “less than 1 h,” “1–3 h,” “3–5 h,” and “more than 5 h.” Categories 1–3 h and 3–5 h were merged into one category based on the distribution of the responses in each category for the analysis.

#### 2.3.3. Coronavirus anxiety scale

The Coronavirus Anxiety Scale (CAS) is a 5-item self-reported mental health screening tool designed to identify probable causes of dysfunctional anxiety associated with the COVID-19 crisis (6). Participants are asked to report on the frequency of specific experiences (i.e., “I felt dizzy, lightheaded, or faint when I read or listened to news about the coronavirus”) over the preceding 2 weeks. The frequency of each item is rated on a 5-point Likert scale, and total scores range from 0 to 20. Based on the CAS score distribution in our sample (mean = 2.3; SD = 3.5), we dichotomized CAS scores ≤1 as “little to no COVID-19-related anxiety” and CAS > 1 as “some COVID-19-related anxiety.” Independent studies of adults in US have demonstrated that the CAS is a reliable instrument (*α*s > 0.90), with solid factorial (single-factor; invariant across sociodemographic) and construct (correlated with anxiety, depression, suicidal ideation, and drug/alcohol coping) validity. A CAS total score ≥ 9 indicates probable dysfunctional coronavirus-related anxiety. Elevated scores on a particular item or a high total scale score (≥9) may indicate problematic symptoms for the individual that might warrant further assessment and/or treatment (6).

### 2.4. Analysis

Descriptive statistics were conducted to examine the binary relationships between demographic factors (i.e., age, sex, and ethnicity), screen time (i.e., passive watching, social media, education, and video games) and COVID-19-related anxiety. Also, partially adjusted and fully adjusted binary logistic regression analyses were conducted to examine the association between the types of screen time and COVID-19-related anxiety. All analyses were conducted using Software for Statistics and Data Science (STATA; version 16.0).

## 3. Results

Approximately half of the youth (47.9%, *N* = 56) used social media over 3 h per day during the pandemic. Slightly over half of the youth (61.5%, *N* = 72) youth of our sample spent over 3 h per day engaging in passive screen time. Most of our youth (82.6%, *N* = 98) spent over 3 h per day on the screens for educational purposes and spent the least time on video games (23.9%, *N* = 28). Table 1 presents COVID-19-related anxiety and screen time based on participant characteristics. Females tended to engage in more hours per day of passive, social media, and educational screen time but less videogame screen time, than males. White youth, on average, engaged in more passive and educational screen time than non-White youth. Young adults and adolescents reported similar screen time exposure, except for social media, which was higher among young adults.

**Table 1:** COVID-19-related anxiety and screen time based on participant characteristics (*N*, %).

	Sex		Ethnicity		Age group	
	Female (*N* = 87)	Male (*N* = 24)	White (*N* = 90)	Non-White (*N* = 20)	Adolescent (*N* = 78)	Young adult (*N* = 39)
CAS (mean, SD)	2.5 (3.4)	1.7 (3.4)	2.4 (3.6)	2.1 (3.4)	2.0 (3.3)	3.0 (3.7)
Screen time						
Passive						
<1 h	3 (3%)	2 (8%)	5 (6%)	1 (5%)	4 (5%)	2 (5%)
1–5 h	49 (56%)	15 (63%)	48 (53%)	14 (70%)	45 (58%)	23 (59%)
>5 h	35 (40%)	7 (29%)	37 (41%)	5 (25%)	29 (37%)	14 (36%)
Social media						
<1 h	17 (20%)	10 (42%)	20 (22%)	7 (35%)	21 (27%)	6 (2%)
1–5 h	39 (45%)	11 (46%)	43 (48%)	6 (30%)	36 (46%)	19 (49%)
>5 h	31 (36%)	3 (13%)	27 (30%)	7 (35%)	21 (27%)	14 (36%)
Video games						
<1 h	46 (53%)	5 (21%)	40 (44%)	12 (60%)	33 (42%)	20 (51%)
1–5 h	34 (39%)	14 (58%)	40 (44%)	6 (30%)	35 (45%)	17 (44%)
>5 h	7 (8%)	5 (21%)	10 (12%)	2 (10%)	10 (13%)	2 (5%)
Education						
<1 h	9 (10%)	0 (0%)	6 (7%)	2 (10%)	7 (9%)	2 (5%)
1–5 h	26 (30%)	13 (54%)	28 (31%)	8 (40%)	27 (35%)	14 (36%)
>5 h	52 (60%)	11 (46%)	56 (62%)	10 (50%)	44 (56%)	23 (59%)

CAS, Coronavirus anxiety scale.

**Figure 1:**
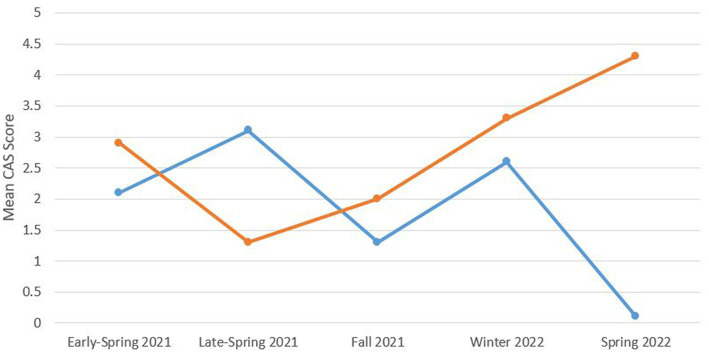
Adolescent (blue) and young adult (orange) mean CAS scores across the recruitment cohort. CAS, Coronavirus anxiety scale.

In terms of COVID-19-related anxiety, average CAS scores were 2.3 (SD = 3.5), and 62% of youths reported little to no COVID-19-related anxiety (CAS ≤ 1). However, 11.4% of the youth in our sample had scores that met a clinically significant threshold of nine (6). Table 2 summarizes screen time and CAS scores across all five recruitment cohorts to understand better the impact of the evolving landscape of the COVID-19 pandemic. We observed an increase in CAS scores and educational screen time in Late-Spring 2021. Figure 1 shows an interaction of CAS scores with age groups across the recruitment cohort. Interestingly, young adult CAS scores were lower during the highly restrictive Late-Spring 2021 cohort but were considerably higher in our Spring 2022 cohort.

**Table 2:** Provincial restrictiveness, COVID-19 anxiety, and screen time usage according to recruitment cohort.

	2021			2022	
	Early-Spring (*N* = 30)	Late-Spring (*N* = 19)	Fall (*N* = 15)	Winter (*N* = 30)	Spring (*N* = 20)
Variant of concern	Gamma	Gamma	Delta	Omicron	Omicron
School closures	No	Yes	No	Yes	No
Provincial lockdown	No	Yes	No	No	No
Masks mandatory	Yes	Yes	Yes	Yes	No
Received 1st vaccine^a^	11.3%	65.3%	75.5%	82.9%	84.5%
Received 2nd vaccine^a^	2.1%	19.4%	70.6%	77.5%	81.4%
Received 1st vaccine^b^	0%	51.9%	81.5%	87.3%	88.1%
Received 2nd vaccine^b^	0%	2.2%	71.9%	82.9%	84.3%
CAS	2.3 (3.9)	2.5 (3.1)	1.5 (2.0)	2.8 (4.1)	2.0 (3.5)
Screen time					
Passive					
<1 h	0 (0%)	2 (10%)	1 (7%)	1 (3%)	2 (10%)
1–5 h	19 (63%)	11 (55%)	9 (60%)	21 (66%)	8 (40%)
>5 h	11 (37%)	7 (35%)	5 (33%)	10 (31%)	10 (50%)
Social media					
<1 h	10 (33%)	4 (20%)	4 (27%)	5 (16%)	4 (20%)
1–5 h	15 (50%)	7 (35%)	5 (33%)	20 (63%)	8 (40%)
>5 h	5 (17%)	9 (45%)	6 (40%)	7 (22%)	8 (40%)
Video games					
<1 h	15 (50%)	11 (55%)	8 (53%)	10 (31%)	9 (45%)
1–5 h	12 (40%)	8 (40%)	7 (47%)	16 (50%)	9 (45%)
>5 h	3 (10%)	1 (5%)	0 (0%)	6 (19%)	2 (10%)
Education					
<1 h	3 (10%)	0 (0%)	3 (20%)	2 (6%)	1 (5%)
1–5 h	12 (40%)	6 (30%)	4 (27%)	12 (38%)	7 (35%)
>5 h	15 (50%)	14 (70%)	8 (53%)	18 (56%)	12 (60%)

Source: https://health-infobase.canada.ca/covid-19/vaccination-coverage/.

CAS, Coronavirus anxiety scale.

a Adults > 17.

b Adolescents age 12–17.

In a partially adjusted model (accounting for other types of screen time), 1–5 daily hours on social media was associated with increased odds of experiencing COVID-19-related anxiety compared to those who spend less than 1 h per day (OR = 3.50, 95% CI = 1.14–10.72, *p* < 0.05). Other types of screen time were not significantly associated with COVID-19-related anxiety. In a fully adjusted model (accounting for age, sex and ethnicity besides four types of screen time), 1–5 h of social media remained significantly associated with COVID-19-related anxiety (OR = 4.08, 95% CI = 1.22–13.62, *p* < 0.05). Although the positive direction of the association between social media >5 and CAS aligns with the association between 1–5 h and CAS, the association between social media >5 and CAS was not significant. Wide confidence intervals in regression models may indicate that the power to declare the differences is low and may lead to a type I error. We suspect that the relatively smaller sample size for social media >5 h (*N* = 35) than the 1–5 h (*N* = 55) may have limited this non-significant social media effect on CAS, as demonstrated in the wide 95% Confidence intervals in model 1 and 2 (M1: OR = 2.78, 95% CI = 0.77–10.05; M2: OR = 1.85, 95% CI = 0.46–7.44; see Table 3).

**Table 3:** Associations between screen time and participant characteristics and COVID-19-related anxiety (Odds ratio, 95% CI).

	Partially adjusted model (Model 1)	Fully adjusted model (Model 2)
Screen time		
Passive		
<1 h	ref	ref
1–5 h	0.66 (0.92–4.71)	0.44 (0.03–5.77)
>5 h	0.47 (0.06–3.42)	0.17 (0.01–2.26)
Social media		
<1 h	ref	ref
1–5 h	3.50 (1.14–10.72)*	4.08 (1.22–13.62)*
>5 h	2.78 (0.77–10.05)	1.85 (0.46–7.44)
Education		
<1 h	ref	ref
1–5 h	2.00 (0.25–16.11)	2.15 (0.24–19.45)
>5 h	1.21 (0.17–8.74)	1.55 (0.19–12.62)
Video game		
<1 h	ref	ref
1–5 h	0.48 (0.20–1.15)	0.45 (0.16–1.22)
>5 h	0.71 (0.17–2.89)	0.94 (0.20–4.51)
Age		
Adolescent (12–17 yrs. old)		ref
Young adult (18–24 yrs. old)		2.40 (0.82–7.01)
Sex		
Female		ref
Male		0.42 (0.12–1.52)
Ethnicity		
White		ref
Non-white		1.01 (0.32–3.25)

* <0.05; Confounding variables: Age, sex, and ethnicity.

## 4. Discussion

We investigated the daily screen time usage and COVID-19-related anxiety during the pandemic among Canadian youth and probed the association between the four types of screen time and COVID-19-related anxiety. Most youths reported spending over 3 h per day on screens for pleasure (passive screen time and social media) and educational purposes during the pandemic. COVID-19-related anxiety was the highest during late spring 2021, and 11.4% of the participants reported experiencing a dysfunctional level of anxiety for COVID-19. Further, frequent social media use was positively associated with COVID-19-related anxiety.

Our findings align with the growing body of evidence suggesting heightened daily screen time use during the pandemic among youth (8–12), while less than 2 h of daily screen time for pleasure is recommended by the Canadian 24 h movement guideline (23). Only 36.2% of post-secondary students (23) and 8% of children and adolescents (24) in Canada adhered to the Canadian 24 h movement guidelines for screen time. Most of our youths engaged in daily screen time over 3 h, exceeding the Canadian 24 h movement guidelines (23). Among them, 36.8% reported spending more than 5 hours daily on social media. Heightened daily screen time implicates lifestyle disruption, including decreased physical activity during the pandemic (10, 24).

Further, approximately one-third (38%) of youth reported experiencing some degree of COVID-19-related anxiety, and 11.4% of our sample had coronavirus-related anxiety scores that met a clinically significant threshold of nine, indicating a probable dysfunctional coronavirus-related anxiety (6). Our findings corroborate youth’s pandemic-related concerns, such as their anxiety about the virus itself (e.g., fear of getting COVID-19, feeling unwell and unsafe) (17). The COVID-19-related anxiety was highest in late spring 2021 when provincial mandates were the most stringent. For instance, the provincial public health measure included school closure (April 19 to June 28, 2021) and a “stay-at-home” order (April 8 to June 2, 2021) that restricted all travel outside the house unless deemed essential (i.e., for work, to purchase groceries, for healthcare) during late spring 2021. Amidst these restrictions, only half of the eligible Ontarians between the ages of 12 and 17 had received their first COVID-19 vaccine, and less than 5% were fully vaccinated (received at least two COVID-19 vaccines). Given the circumstances during Late-Spring 2021, it is not surprising that we observed an increase in COVID-19-related anxiety, social media and educational screen time compared to the cohorts recruited prior to and after, Late-Spring 2021. Also, young adults showed heightened COVID-19-related anxiety than adolescents in Spring 2022. Speculatively, this age group contrast for COVID-19-related anxiety in Spring 2022 reflects the return to in-person learning for most post-secondary students in Ontario. For example, COVID-19-related policies/guidelines for post-secondary institutions were less harmonized, and many post-secondary students felt uncertain and were concerned about their health, mental health, and academic futures (25).

So far, this increase in screen time in youth, particularly when engaging with social media use during the pandemic (16, 18, 21), has been associated with poorer mental health outcomes (8, 12, 16–20). Our findings uniquely add to the literature by suggesting that accounting for other types of screen time and demographic information, spending 1–5 h daily on social media is associated with experiencing COVID-19-specific anxiety. A large volume of COVID-19-related topics is spread/shared on social media platforms, including misinformation from unreliable sources, creating “the COVID-19 social media infodemic” (26). Further, young people often recounted anxiety about COVID-19’s impact on their wellbeing via social media (17). For example, a real-time, multi-platform online ethnography from March 2020 to March 2021 revealed that youth often commented on mental health difficulties arising from public safety measures and social disconnectedness and recounted their anxiety about the virus’s impact on others (17). Further, although adolescents who feel lonely tended to use more social media during the pandemic, presumably to cope with the lack of social contact, it did not help them feel happier (27). We speculate that significant information sharing (from reliable and unreliable sources) related to the pandemic was found to have occurred on various social media platforms such as Twitter, Instagram, YouTube, Reddit, and Gab, escalating anxiety among social media users.

Given this evidence, a growing need exists to consider different strategies to promote healthy screen hygiene in youth, particularly on social media during the pandemic and beyond.

Firstly, evidenced-based strategies have been reported that promote healthy social media screen time for youth to mitigate mental health harm, such as increased COVID-19 anxiety. The Canadian 24-Hour Movement Guidelines for Children and Youth recommend no more than 2 h per day of recreational screen time (23). To limit the negative impact of social media screen time, parents and caregivers may promote healthy social media screen use, encourage screen-free time, and limit anxiety-provoking content such as excessive pandemic coverage. Parents and caregivers can also be encouraged to model healthy social media screen hygiene by limiting their social media screen time (28). Guidance and support may be required to assist parents and caregivers in supporting youth to limit social media screen time through educational, public health, or health promotion campaigns (29).

Secondly, while parents and caregivers play an important role, they cannot be expected to address social media screen time increases alone. As such, a broader policy response may be warranted. Prior to the pandemic, youth remained connected with their peers through various activities such as in-person schooling, extracurricular programming, and online through various social media platforms. Due to public health restrictions, social media has allowed youth to stay connected to friends and family, especially when school and recreational facilities were closed (29). However, prolonged and excessive social media screen time use has also been linked to negative mental health outcomes (16, 18, 21). Prioritizing in-person schooling and extracurricular programming by ensuring a safe environment that supports uninterrupted full-time learning for youth may reduce social media screen time and endorse healthy behaviours at an important developmental stage.

Lastly, it is warranted to invest in resources and training opportunities to encourage youth to reflect on social media screen use and its impact on their mental health. Providing training, tools and strategies to navigate the online environment safely may promote healthy youth development (28, 30). This could include providing training on screen hygiene and social media screen time safety skills within an educational setting, which may be an effective strategy to reach a broad range of youth regardless of demographic and household factors.

There are several limitations to this current study. The cross-sectional design precludes our ability to understand the temporal ordering between screen time and COVID-19-related anxiety. Also, the sample size (*N* = 117) is modest for the number of variables we included in our regression models (model 1 = 4, model 2 = 7). We followed the rule of thumb to have at least 10 observations per predictor (31). To prove that the sample size (*N* = 117) is appropriate for the regression analysis we conducted to determine the impact of social media on COVID-related anxiety, we used SPSS Version 28 Power Analysis to estimate the *N* needed to detect a small effect (0.20) in regressions with *α* = 0.05, power = 0.8, and 7 total number of predictors (4 test predictor), which returned an estimated *N* of 65. However, wide Confidence Intervals in regression models may indicate that the power to declare the differences is low, leading to a type I error. Replicating the findings with a larger sample, with proportionate number of participants for age, gender and ethnicity, using a longitudinal study design is warranted to further determine the relationship between screen time and COVID-19-related anxiety. Lastly, future studies should include more detailed demographic information of the sample, such as income level, occupation, and education to better characterize the sample and its links to screen time and COVID-related anxiety.

## 5. Conclusion

To our knowledge, this is the first study that explored the role of four screen time types (passive watching, social media, online games, and education) in COVID-19-related anxiety among Canadian youth. Based on our findings, COVID-19-related anxiety was the highest during late spring 2021, during the third wave of the COVID-19 pandemic when provincial mandates were the most stringent. At that time, 11.4% of the participants reported experiencing a dysfunctional level of anxiety for COVID-19. Further, frequent social media use was positively associated with COVID-19-related anxiety. Knowledge gained from this study will guide clinicians, parents, and educators to work collaboratively to provide developmentally appropriate approaches to reduce the negative social media impact on COVID-19-related anxiety among youth as our society recovers from the pandemic.

## Data availability statement

The raw data supporting the conclusions of this article will be made available by the authors, without undue reservation.

## Ethics statement

The studies involving human participants were reviewed and approved by the Waypoint Centre for Mental Health Care Research Ethics Board (HPRA# 21.03.02). All participants provided their written informed consent to participate in this study and legal guardians were informed of their participation.

## Author contributions

All authors provided critical reviews, contributed to the final manuscript and approved the submitted version.

## Funding

This study was supported by an Insight Development Grant from the Social Sciences and Humanities Research Council (SSHRC) awarded to SK (# 430–2020-00288).

## Conflict of interest

The authors declare that the research was conducted in the absence of any commercial or financial relationships that could be construed as a potential conflict of interest.

## Publisher’s note

All claims expressed in this article are solely those of the authors and do not necessarily represent those of their affiliated organizations, or those of the publisher, the editors and the reviewers. Any product that may be evaluated in this article, or claim that may be made by its manufacturer, is not guaranteed or endorsed by the publisher.

## Figures and Tables

**Table A1:** COVID-19-related anxiety (average CAS score) for passive, social media, video games, and education screen time (<1 h, 1–4 h, >5 h per day) in five-time points (Early-Spring 2021, Late-Spring 2021, Fall 2022, Winter 2022, and Spring 2022).

	2021			2022	
	Early-Spring (*N* = 30)	Late-Spring (*N* = 19)	Fall (*N* = 15)	Winter (*N* = 30)	Spring (*N* = 20)
Passive	% (CAS)				
<1 h	0%	10% (2.50)	7% (0.00)	3% (1.00)	10% (4.50)
1–5 h	63% (2.58)	55% (2.18)	60% (2.22)	66% (3.58)	40% (2.50)
>5 h	37% (1.82)	35% (3.17)	33% (0.40)	31% (1.60)	50% (1.10)
Social media					
<1 h	33% (1.40)	20% (3.46)	27% (1.25)	16% (2.80)	20% (0.00)
1–5 h	50% (2.13)	35% (3.93)	33% (2.00)	63% (3.39)	40% (2.50)
>5 h	17% (4.60)	45% (2.28)	40% (1.17)	22% (1.43)	40% (2.50)
Video games					
<1 h	50% (2.33)	55% (3.34)	53% (2.50)	31% (3.10)	45% (2.33)
1–5 h	40% (1.80)	40% (2.71)	47% (0.29)	50% (2.50)	45% (2.11)
>5 h	10% (4.33)	5% (0.00)	0%	19% (3.17)	10% (0.00)
Education					
<1 h	10% (0.33)	0% (2.00)	20% (1.67)	6% (0.00)	5% (3.00)
1–5 h	40% (2.17)	30% (2.77)	27% (2.75)	38% (2.60)	35% (1.29)
>5 h	50% (2.80)	70% (2.53)	53% (0.75)	56% (3.28)	60% (2.33)

CAS, Coronavirus anxiety scale.
